# A rare neuroendocrine tumor of the lung

**DOI:** 10.11604/pamj.2023.46.54.41283

**Published:** 2023-10-13

**Authors:** Ashwin Karnan

**Affiliations:** 1 Department of Respiratory Medicine, Jawaharlal Nehru Medical College, Datta Meghe Institute of Higher Education and Research, Sawangi (Meghe), Wardha, Maharashtra, India

**Keywords:** Cough, neuroendocrine, immunohistochemistry, lung biopsy, small cell carcinoma

## Image in medicine

A 51-year-old female presented to us with complaints of breathlessness, cough with expectoration, left-sided chest and back pain with general tiredness for the past 1 month. She was a farmer by profession, with no significant past history. All routine investigations were done. Contrast computed tomography thorax showed a large heterogeneously enhancing soft tissue mass occupying the left hemithorax and single precarinal necrotic node. Bronchoscopy followed by CT-guided lung biopsy was done, which showed a neuroendocrine tumour with high mitotic activity and extensive necrosis suggestive of large cell neuroendocrine carcinoma. Immunohisto chemical staining for neuroendocrine markers was done, which was positive for chromogranin A and synaptophysin. She was then shifted to the oncology department for surgery followed by chemotherapy. Lung neuroendocrine tumours are rare tumours accounting for about 20% of all lung tumours, 1-2 % of all tumours and 25% of all neuroendocrine tumours. Lung tumours comprise 75-80%, neuroendocrine tumours (NETs), 1-2 % carcinoid tumours (typical and atypical carcinoid), 3% large cell neuroendocrine carcinoma of the lung (LCNEC) and 15-20% small cell lung cancer (SCLC). Immunohistochemical examination is the most important criterion for lung neuroendocrine tumours (LNET). The classical symptoms of carcinoid tumours are cough, dyspnoea, recurrent respiratory tract infection and haemoptysis. For patients with high surgical risk, interventional bronchoscopy and endobronchial resection may be done. For advanced SCLC, chemotherapy with cisplatin and etoposide is the standard treatment.

**Figure 1:**
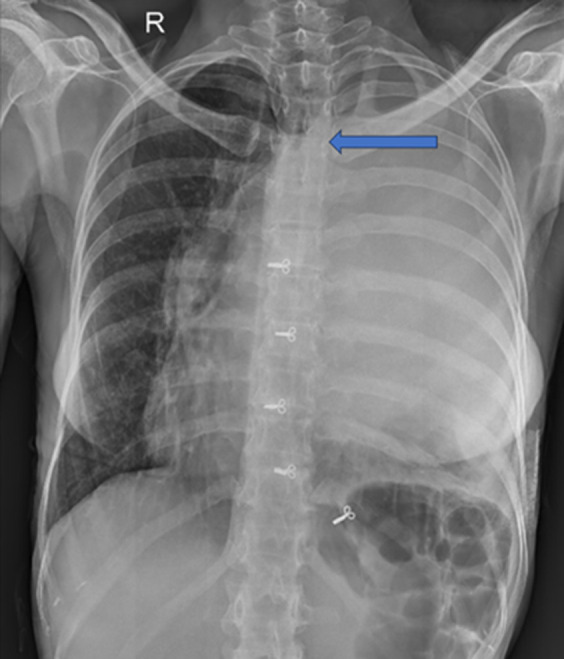
chest X-ray showing left opaque hemithorax with trachea pushed to the right side and a blue arrow showing left bronchus cut-off sign

